# Open‐Shell Conjugated Polymers Enable Photothermoelectric Conversion and Detection of Short‐Wave Infrared Light

**DOI:** 10.1002/advs.77195

**Published:** 2026-08-19

**Authors:** Guan‐Lin Chen, Soonyong Lee, Shao‐Huan Hong, Jhih‐Min Lin, Han Young Woo, Cheng‐Liang Liu

**Affiliations:** ^1^ Department of Materials Science and Engineering National Taiwan University Taipei Taiwan; ^2^ Department of Chemistry Korea University Seoul Republic of Korea; ^3^ National Synchrotron Radiation Research Center Hsinchu Taiwan; ^4^ Advanced Research Center for Green Materials Science and Technology National Taiwan University Taipei Taiwan

**Keywords:** detector, open‐shell structure, photothermoelectric, short‐wave infrared

## Abstract

Short‐wave infrared (SWIR) light constitutes a significant yet underutilized portion of the solar spectrum, and efficient harvesting and detection of SWIR photons are increasingly required for emerging photonic and energy technologies. Here, we demonstrate SWIR energy harvesting and photodetection using an organic photothermoelectric (OPTE) device based on a proquinoidal diradicaloid‐type conjugated polymer, PBBT2T‐T, which exhibits broadband absorption extending from the visible to the SWIR region due to intrinsic free‐charge carriers. After F4TCNQ doping, the OPTE device delivered an open‐circuit voltage of 329.6 µV, a short‐circuit current of 2.67 µA, and a maximum output power of 0.22 nW under >1000 nm illumination (50 mW cm^−2^). Furthermore, the device functions as an OPTE detector capable of sensing SWIR light beyond 1500 nm, achieving a responsivity of 0.0063 V W^−1^ and a detectivity of 8.4 × 10^5^ Jones. Notably, this result successfully demonstrates SWIR photodetection based on the photothermoelectric effect for the first time. These findings establish OPTE conversion as a promising strategy for SWIR energy harvesting and photodetection, with strong potential to overcome the intrinsic limitations of conventional organic SWIR optoelectronic technologies.

## Graphical abstract

Efficient conversion and detection of short‐wave infrared (SWIR) photons are becoming increasingly important for emerging photonic and energy technologies. Here, we report an organic photothermoelectric device based on an open‐shell conjugated polymer that enables SWIR energy conversion and photon detection via the photothermoelectric effect.

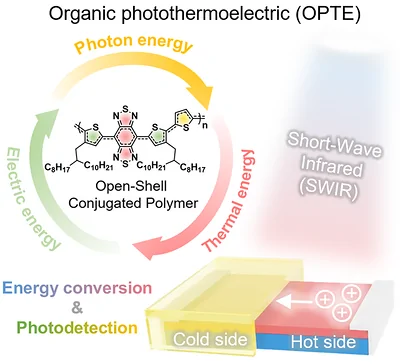

## 1 Introduction

Efficient harvesting and detection of long‐wavelength photons are increasingly important for emerging photonic and energy technologies. In particular, short‐wave infrared (SWIR, 1000–2500 nm) radiation exhibits low optical scattering and strong penetration capability, enabling high signal‐to‐noise ratios even in complex environments such as fog, smoke, or biological tissues [1, 2]. Owing to these advantages, SWIR light has found wide‐ranging applications in remote sensing, night‐vision imaging, biomedical diagnostics, and optical communications [3, 4, 5, 6]. Furthermore, advanced technologies such as light detection and ranging (LiDAR) require highly sensitive and stable photodetection, especially at wavelengths beyond 1500 nm [7]. From an energy perspective, SWIR photons account for nearly one‐third of the total solar irradiance (Figure S1), representing a substantial yet largely underutilized energy resource. Consequently, developing materials and device platforms capable of efficiently harvesting and detecting SWIR photons remains an important challenge in both photonics and energy research.

Organic optoelectronic systems offer several advantages for photoconversion technologies, including mechanical flexibility, low‐temperature solution processability, and tunable electronic structures [8, 9, 10, 11, 12, 13, 14, 15]. However, extending the photoresponse of organic devices into the SWIR region remains fundamentally challenging. In conventional organic photovoltaics (OPVs) and organic photodetectors (OPDs), photoconversion relies on exciton generation followed by interfacial exciton dissociation within donor–acceptor bulk heterojunction (BHJ) architectures [16]. Efficient charge separation in such systems critically depends on appropriate energy‐level offsets between donor and acceptor materials. As the target absorption wavelength extends into the SWIR region, the optical bandgap of organic semiconductors must become extremely narrow, which significantly reduces the energetic driving force for exciton dissociation and charge transfer. Consequently, the distinction between donor and acceptor materials becomes increasingly ambiguous, limiting exciton separation, and charge generation efficiency (Figure S2). As a result, the spectral response of most OPV and OPD systems is predominantly confined to the visible and near‐infrared region (300–1000 nm), while efficient photodetection beyond 1500 nm remains particularly challenging (Figure S3 and Table S1).

An alternative strategy to overcome these intrinsic limitations is to utilize the photothermoelectric (PTE) effect, which converts absorbed photons into electrical signals through a light–heat–electricity conversion pathway [17, 18]. Upon photoexcitation, absorbed photon energy is predominantly dissipated through nonradiative relaxation processes, generating localized heat, and establishing a temperature gradient (Δ*T*) within the material [19]. Under open‐circuit conditions, thermoelectric carrier diffusion driven by the Seebeck effect produces a measurable voltage and electrical output [20, 21, 22, 23]. Because the conversion mechanism is governed primarily by photothermal energy rather than exciton dissociation, photothermoelectric systems bypass the stringent requirements of energy‐level matching and charge‐transfer energetics. This feature makes the PTE mechanism particularly attractive for harvesting long‐wavelength photons, including those in the SWIR region.

In this context, organic semiconductors with open‐shell electronic structures represent promising candidates for SWIR photothermal conversion [24, 25, 26]. Open‐shell conjugated polymers can stabilize polaron and bipolaron states, which introduce broad sub‐bandgap absorption extending deep into the near‐ and short‐wave infrared regions [27, 28]. Such polaronic absorption bands can efficiently capture long‐wavelength photons and convert them into heat, thereby enabling effective PTE energy conversion beyond the intrinsic bandgap limit of conventional closed‐shell semiconductors.

Herein, we employ an open‐shell conjugated polymer, PBBT2T‐T, containing a pro‐quinoidal benzo[1,2‐c:4,5‐c′]bis[1,2,5]thiadiazole (BBT) unit, and compare it with a closed‐shell analogue, PBT2T‐T, based on 1,2,5‐benzothiadiazole (BT) (Figure 1a). Owing to its open‐shell electronic structure, PBBT2T‐T exhibits markedly broadened absorption extending from the visible to the SWIR region, which is particularly advantageous for photothermal energy conversion at long wavelengths. Upon molecular doping with F4TCNQ, the resulting organic photothermoelectric (OPTE) device efficiently converts SWIR photons into electrical output via thermally driven carrier diffusion. Under >1000 nm illumination (50 mW cm^−2^), the device delivers an open‐circuit voltage (*V_oc_*) of 329.6 µV, a short‐circuit current (*I_sc_*) of 2.67 µA, and a maximum output power (*P_out_*) of 0.22 nW, while maintaining stable operation for over 36 000 s.

**FIGURE 1:**
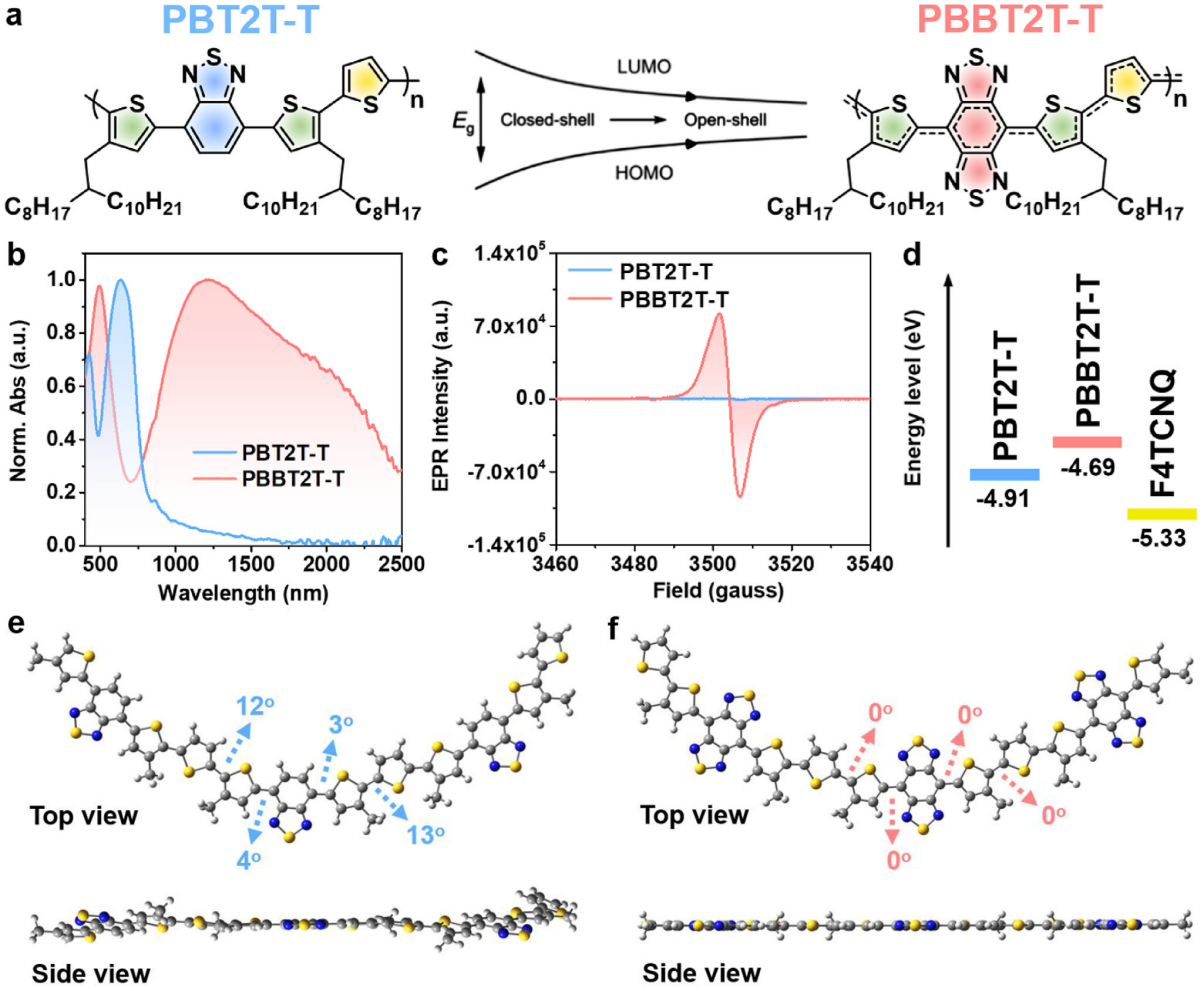
(a) Chemical structures of PBT2T‐T and PBBT2T‐T, along with a schematic illustration of the bandgap narrowing from a closed‐shell structure to an open‐shell diradical character. (b) UV–vis–NIR absorption spectra of PBT2T‐T and PBBT2T‐T films. (c) Room‐temperature EPR spectra of PBT2T‐T and PBBT2T‐T. (d) Energy level diagram of PBT2T‐T, PBBT2T‐T, and F4TCNQ. Top and side views of the dihedral angles in the DFT‐optimized molecular structures of (e) PBT2T‐T and (f) PBBT2T‐T.

Moreover, the OPTE device functions as an organic photothermoelectric detector (OPTED) capable of detecting SWIR light beyond 1500 nm, achieving a responsivity of 0.0063 V W^−1^ and a detectivity of 8.4 × 10^5^ Jones with stable performance over more than 100 on/off cycles. To the best of our knowledge, this work represents the first demonstration of SWIR photodetection based on the OPTE effect. These results highlight the unique advantages of open‐shell conjugated polymers for long‐wavelength photoconversion and establish PTE conversion as a promising strategy to overcome the intrinsic limitations of conventional organic SWIR energy‐harvesting devices and photodetectors.

## 2 Results and Discussion

Diradicaloids exhibit partial open‐shell character, placing their electronic structures between conventional closed‐shell molecules and true radicals [29, 30]. These systems typically feature ultranarrow bandgaps (0.1 eV < E_g_ < 1 eV), where bandgap narrowing induces strong configurational mixing between the highest occupied and lowest unoccupied molecular orbitals (HOMO–LUMO) [31]. Consequently, the frontier electronic structure evolves toward nearly degenerate singly occupied molecular orbitals (SOMOs), enabling the emergence of unpaired electrons and open‐shell character [32]. The radical character originates from the synergistic interplay of multiple structural and electronic factors, including intramolecular electron delocalization, conjugation length, donor–acceptor push–pull interactions, and aggregation‐induced radical (AIR) effects [33, 34]. Together, these factors govern both the stability and population of the diradical state.

Importantly, open‐shell diradicaloid conjugated systems generally exhibit strong and broad optical absorption extending into the NIR and SWIR regions [35, 36]. Furthermore, the open‐shell ground state can promote the spontaneous formation of delocalized radical species or intrinsic charge carriers along the π‐conjugated backbone [37]. As a result, relatively high electrical conductivity can be achieved even in the absence of external chemical doping [38]. The presence of these intrinsic free carriers facilitates efficient charge transport and also enhances nonradiative relaxation pathways, enabling effective conversion of absorbed photons into thermal energy [24, 25, 26]. These characteristics make open‐shell diradicaloid conjugated polymers particularly attractive for OPTE systems, where efficient photothermal conversion and carrier transport are essential for harvesting and detecting long‐wavelength SWIR photons.

An open‐shell diradicaloid polymer, PBBT2T‐T, and its closed‐shell counterpart, PBT2T‐T, were synthesized according to previously reported procedures (Scheme S1) [38, 39]. PBBT2T‐T was prepared in 32.4% yield via Stille coupling of 4,8‐bis(5‐bromo‐4‐(2‐octyldodecyl)thiophen‐2‐yl)benzo[1,2‐c;4,5‐c′]bis[1,2,5]thiadiazole (M2) and 2,5‐bis(trimethylstannyl)thiophene (M3) using tris(dibenzylideneacetone)dipalladium(0) as the catalyst in anhydrous xylene. PBT2T‐T was synthesized analogously through Stille coupling of 4,7‐bis(5‐bromo‐4‐(2‐octyldodecyl)thiophen‐2‐yl)benzo[c][1,2,5]thiadiazole (M1) and M3, yielding the polymer in 61.3%. The resulting polymers were purified by Soxhlet extraction using a sequence of solvents. Their chemical structures were confirmed by ^1^H NMR spectroscopy (Figures S4–S7). The molecular weights and dispersities of the polymers are summarized in Table S2. Thermogravimetric analysis (TGA) revealed high thermal stability for both polymers, with 5% weight‐loss temperatures exceeding 350°C (Figure S8a). Differential scanning calorimetry (DSC) showed melting transitions at 114°C for PBT2T‐T and 297°C for PBBT2T‐T, indicating the higher backbone rigidity and thermal robustness of PBBT2T‐T (Figure S8b).

The optical properties of the polymers were investigated using UV–vis–NIR spectroscopy (Figure 1b). Notably, PBBT2T‐T displays broad and intense absorption extending from 400 nm deep into the SWIR region, with a π–π* transition centered at 496 nm and a pronounced low‐energy absorption maximum at 1197 nm. This remarkably red‐shifted and broadened absorption is indicative of extended π‐conjugation and pronounced open‐shell diradical character. In contrast, PBT2T‐T exhibits a narrower absorption profile with a π–π* peak at 422 nm and an intramolecular charge‐transfer (ICT) band centered at 681 nm [40, 41], while the absorption onset at approximately 815 nm reflects a relatively larger optical bandgap consistent with its closed‐shell electronic structure. The maximum absorbance of PBBT2T‐T and PBT2T‐T thin films with different thicknesses was measured (Figure S9a), and linear relationships between maximum absorbance and film thickness were established (Figure S9b). The absorption coefficients were calculated using Beer–Lambert's law (Equation S1), yielding values of 4.2 × 10^4^ cm^−1^ and 7.1 × 10^4^ cm^−1^ for PBT2T‐T and PBBT2T‐T, respectively, indicating that PBBT2T‐T exhibits stronger light absorption capability. To further probe the electronic ground states of the polymers, electron paramagnetic resonance (EPR) spectroscopy was performed at room temperature (Figure 1c). PBBT2T‐T exhibits a significantly stronger EPR signal compared to PBT2T‐T, indicating a much higher density of unpaired spins. This result provides direct evidence for the open‐shell nature of PBBT2T‐T and is consistent with its extended SWIR absorption and reduced bandgap observed in the optical spectra.

Photoelectron spectroscopy in air (PESA) determine HOMO levels of −4.91 eV for PBT2T‐T and −4.69 eV for PBBT2T‐T (Figure S10), with the corresponding energy level diagram shown in Figure 1d. Density functional theory (DFT) calculations, performed using B3LYP/6‐31G(d, p) [42], revealed that PBT2T‐T and PBBT2T‐T have bandgaps of 1.86 and 0.74 eV, respectively (Figure S11). The shallower HOMO and significantly narrower bandgap of PBBT2T‐T are attributed to strong quinoidal resonance and enhanced π‐electron delocalization along the conjugated backbone. Torsional angle analysis (Figure 1e,f) shows that PBT2T‐T adopts a more twisted backbone, whereas PBBT2T‐T exhibits nearly planar conformation, which favors stronger π–π stacking interactions and intermolecular electronic coupling. In addition, molecular electrostatic potential (ESP) mapping (Figure S12) further indicates that PBBT2T‐T possesses a more uniform and continuous charge distribution, which is beneficial for long‐range charge transport.

Molecular doping is an effective strategy to substantially increase the carrier concentration of conjugated polymers, thereby efficiently enhancing both electrical conductivity and PTE power output [43, 44, 45, 46, 47, 48]. Here, F4TCNQ was selected as the dopant due to its high electron affinity of 5.33 eV, enabling efficient p‐type doping through electron extraction from the polymer backbone (Figure 1d) [49, 50]. Vapor‐phase doping was employed, in which F4TCNQ was heated to its sublimation temperature to ensure controlled dopant incorporation into the polymer films (Figure S13). After doping, the primary absorption peaks of both polymers exhibited slight red shifts and partial bleaching, indicating that a fraction of the neutral polymer chains had been converted into charged species (Figure 2a,b). In doped PBT2T‐T, new absorption bands emerged at 800–1000 nm and 1000–2500 nm [51]. The former corresponds to NIR transitions resulting from electronic excitation between lower and higher energy levels within the mid‐gap states, while the latter corresponds to SWIR transitions arising from electron excitation from the HOMO level to the mid‐gap states, representing the formation of (bi)polaron states [52]. Notably, doped PBBT2T‐T exhibits much stronger absorption for the (bi)polaron states compared to doped PBT2T‐T.

**FIGURE 2:**
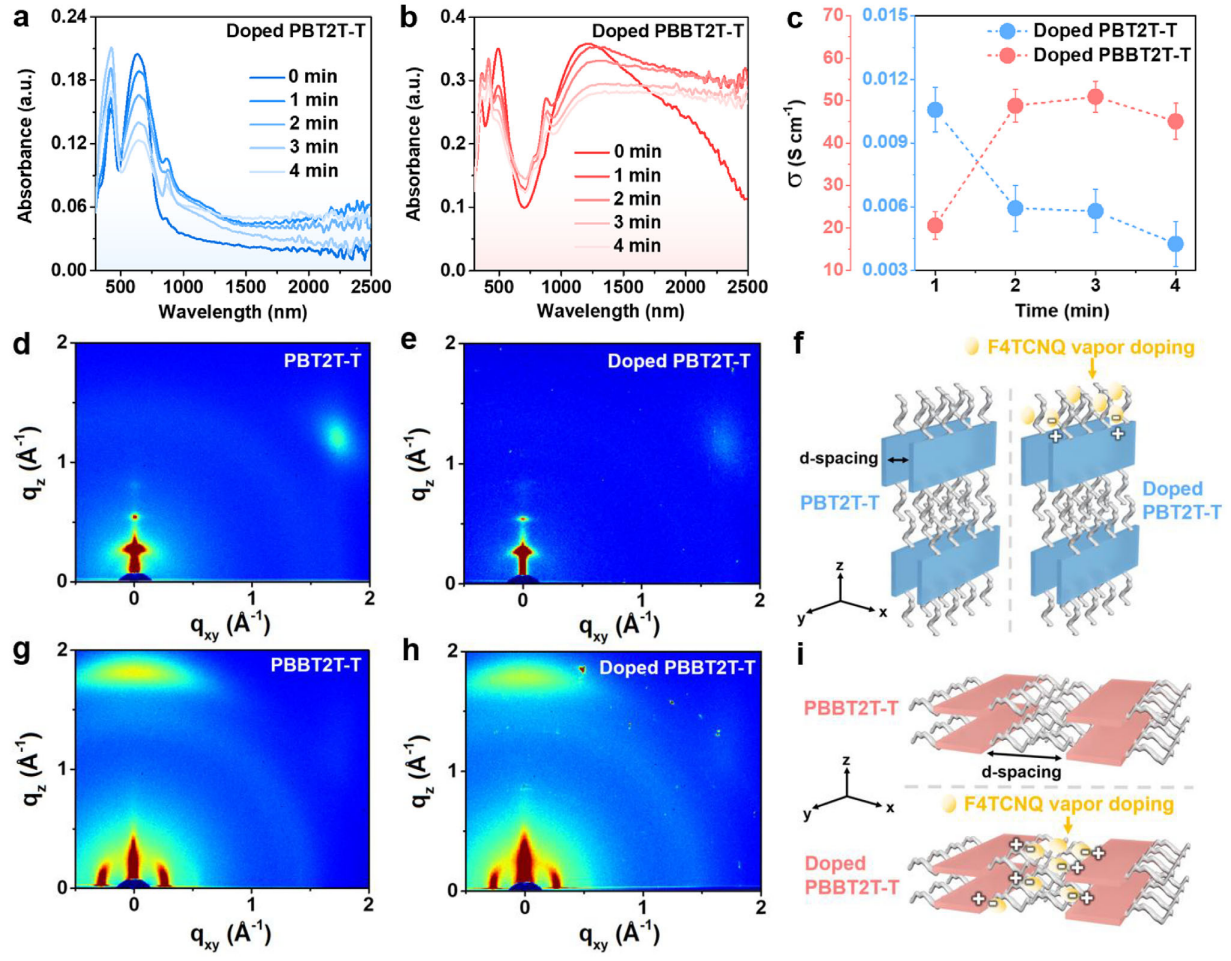
UV–vis–NIR absorption spectra of pristine and doped films of (a) PBT2T‐T and (b) PBBT2T‐T. (c) Electrical conductivity of PBT2T‐T and PBBT2T‐T films vs. vapor‐doping time. 2D‐GIWAXS patterns of polymer films: (d, e) pristine and doped PBT2T‐T and (g, h) pristine and doped PBBT2T‐T films. Illustration of intermolecular orientation and vapor doping of (f) PBT2T‐T and (i) PBBT2T‐T films.

The optimal doping times were 1 min for PBT2T‐T and 3 min for PBBT2T‐T (Figure 2c). Under these conditions, the conductivities reached 0.01 ± 0.001 S cm^−1^ and 50.9 ± 2.6 S cm^−1^, respectively, representing nearly a 5000‐fold increase for PBBT2T‐T. Hall measurements (Figure S14) indicate that this dramatic difference arises primarily from variations in carrier concentration rather than mobility. Specifically, carrier concentrations of doped PBT2T‐T and PBBT2T‐T were 2.02 × 10^16^ cm^−3^ and 6.92 × 10^20^ cm^−3^, respectively, with mobilities of 0.26 and 0.43 cm^2^ V^−1^ s^−1^, demonstrating that PBBT2T‐T possesses a carrier density roughly four orders of magnitude higher.

Room‐temperature EPR spectra (Figure S15) corroborate that the radical signal of doped PBBT2T‐T shows approximately 500 times stronger than that of doped PBT2T‐T, consistent with a significantly higher density of oxidized species. PESA measurements (Figure S16) show that the HOMO levels shifted to −4.95 eV (doped PBT2T‐T) and −5.02 eV (doped PBBT2T‐T), corresponding to downward shifts of 0.04 and 0.33 eV upon F4TCNQ treatments, indicating more efficient p‐doping in PBBT2T‐T. Temperature‐dependent conductivity was fitted using the Arrhenius relation (Equation S2), yielding activation energies of 217.4 meV for doped PBT2T‐T and 58.2 meV for doped PBBT2T‐T (Figure S17) [39]. The lower activation energy of PBBT2T‐T suggests reduced thermal barriers for carrier hopping, consistent with its higher carrier concentration, and more effective charge delocalization.

X‐ray photoelectron spectroscopy (XPS) provides further insights into the doping behavior. The S 2p peak shift of doped PBBT2T‐T (0.38 eV) is significantly larger than that of PBT2T‐T (0.05 eV) upon doping, indicating stronger electron transfer between the polymer backbone and the dopant molecules (Figure S18). The N 1s spectra reveal components corresponding to integer charge transfer (ICT, N^−1^) and charge‐transfer complexes (CTC, N^0^) (Figure S19) [53, 54]. The doping efficiencies (*η_d_*), calculated from peak areas (Equation S3) [44, 50], are 19% for PBT2T‐T and 54% for PBBT2T‐T, clearly demonstrating that the open‐shell polymer exhibits superior doping efficiency.

Atomic force microscopy (AFM) showed that pristine films exhibited smooth nanofibrillar morphologies, whereas doped films displayed nanoscale F4TCNQ aggregates distributed on the surface (Figure S20). 2D‐grazing‐incidence wide‐angle X‐ray scattering (2D‐GIWAXS) measurements revealed that PBT2T‐T adopts an edge‐on orientation both before and after doping, with a lamellar spacing of approximately 22.1 Å and negligible structural changes (Figure 2d,e). In contrast, PBBT2T‐T maintained a face‐on orientation, with the lamellar spacing increasing from 23.2 to 24.3 Å upon doping, while the π–π stacking distance remained unchanged at 3.4 Å (Figure 2g,h). These results suggest that dopants primarily penetrate into and reside within the lamellar regions without disrupting the π‐conjugated backbone packing. Notably, the distinct molecular orientations of the two polymers provide a structural basis for understanding the differences in doping efficiency discussed above. Figure S21 presents the corresponding 1D GIWAXS scattering profiles, and the quantitative peak parameters are summarized in Table S3.

Overall, the edge‐on alignment of PBT2T‐T largely prevents dopant penetration into the film interior. (Figure 2f). In contrast, the face‐on orientation of PBBT2T‐T provides more open lamellar channels, facilitating efficient vapor‐phase diffusion of F4TCNQ (Figure 2i). This favorable molecular packing enables deeper and more effective doping, resulting in a substantially higher carrier density and electrical conductivity. In comparison, doped PBT2T‐T exhibits low doping efficiency and limited carrier concentration, leading to poor electrical conductivity. Therefore, all subsequent studies were conducted exclusively on doped PBBT2T‐T.

The PTE effect proceeds through two sequential processes: first, the material absorbs photons and converts the excitation energy into heat, generating a temperature gradient, which constitutes the photothermal conversion performance; second, the resulting thermal gradient drives carrier diffusion through the Seebeck effect, producing an electrical voltage and power output. Accordingly, we first evaluated the photothermal conversion performance of pristine PBBT2T‐T films under SWIR illumination (λ > 1000 nm), using pristine PBT2T‐T films, which do not absorb SWIR light, as a control. The experimental setup comprised a solar simulator, an aperture mask, a long‐pass optical filter with a 1000 nm cutoff, and a light‐shield cover (schematic in Figure 3a, photograph in Figure S22). During measurement, the light‐shield cover was removed and the surface temperature of the films under SWIR irradiation was continuously monitored using an infrared thermal imaging camera integrated into a custom‐built system. After 30 s of SWIR irradiation at 50 mW cm^−2^, the surface temperatures of pristine PBT2T‐T and PBBT2T‐T films increased by 3.4 and 6.2 K, respectively (Figure S23). The additional 2.8 K temperature increase in pristine PBBT2T‐T is attributed to its broadband SWIR absorption and efficient non‐radiative relaxation pathways, whereas the temperature rise of pristine PBT2T‐T is mainly due to the intrinsic thermal contribution of the incident SWIR radiation. Figure 3b presents infrared thermographs of doped PBBT2T‐T films under different illumination intensities (auto‐scaled), while Figure S24 shows the corresponding time‐dependent temperature profiles. Under SWIR irradiation of 10, 30, and 50 mW cm^−2^ for 30 s, the films exhibited surface temperature increases of 2.0, 3.9, and 6.2 K, respectively.

**FIGURE 3:**
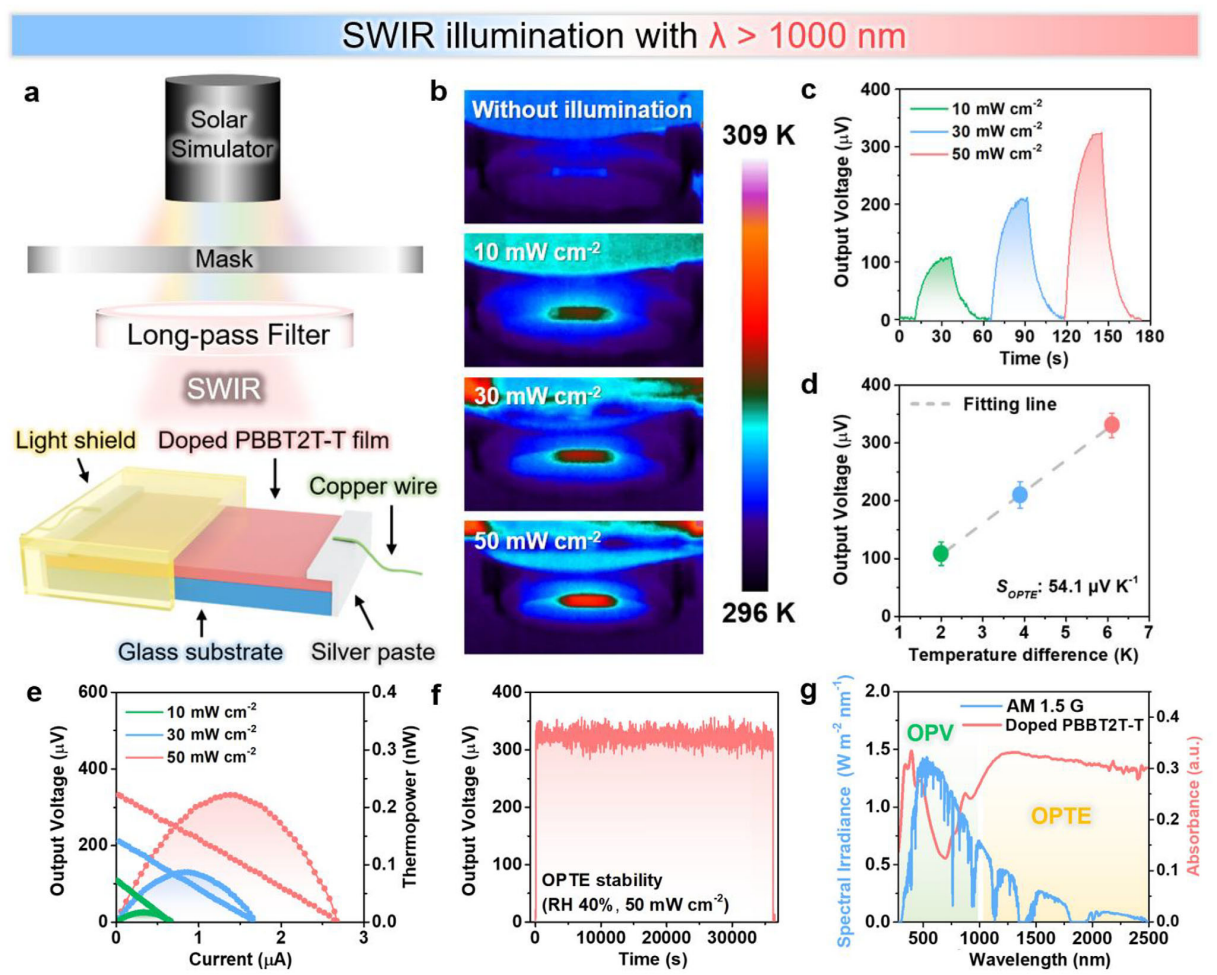
(a) Schematic of the SWIR OPTE measurement setup. (b) IR images and (c) time‐dependent output voltage of the OPTE under varying SWIR (λ >1000 nm) intensities. (d) Linear correlation between output voltage and temperature difference for the OPTE. (e) Voltage and output power vs. current under varying SWIR (λ >1000 nm) intensities. (f) Long‐term output voltage stability under 50 mW cm^−2^ SWIR (λ >1000 nm) illumination at 40% relative humidity. (g) Combined AM1.5G solar spectrum and UV–vis–NIR absorption of doped PBBT2T‐T films.

The organic thermoelectric (OTE) performance was subsequently assessed for films prepared with different vapor‐phase doping durations. Due to the opposing dependence of the Seebeck coefficient (*S_OTE_*) and electrical conductivity (*σ*) on carrier concentration (*n*), an intrinsic trade‐off exists between these parameters (Equation S4). The results indicate that a doping duration of 2 min affords optimal performance (Figure S25). Under these conditions, doped PBBT2T‐T exhibits a *σ* of 52.3 ± 1.1 S cm^−1^, a *S_OTE_* of 54.6 ± 1.4 µV K^−1^, and a power factor (*PF*) of 15.6 ± 0.5 µW m^−1^ K^−2^ (Equation S5), demonstrating that molecular doping effectively enhances carrier density while preserving favorable *S_OTE_* and high power output. All values represent the mean of five independent devices. In terms of long‐term environmental stability, the doped films retained over 85% of their *PF* after 384 h of storage under ambient conditions (25°C and 40% relative humidity, Figure S26a). UV–vis–NIR spectra indicated negligible changes in the (bi)polaron absorption bands (Figure S26b), confirming highly stable oxidized states and effective suppression of dedoping. This remarkable stability is attributed to the synergistic effects of the open‐shell diradical character of PBBT2T‐T, which lowers the HOMO below −5.0 eV after doping to reduce air sensitivity [55], and the chemical inertness of F4TCNQ [53, 56], which stabilizes the charge‐transfer states and suppresses side reactions. These characteristics support the suitability of doped PBBT2T‐T for stable OPTE applications.

The OPTE response of doped PBBT2T‐T films was then evaluated under the optimal vapor doping duration of 2 min, as determined from the OTE measurements. In this configuration, incident SWIR light generates localized heating, producing a temperature gradient between illuminated (hot) and unilluminated (cold) regions of the film. This gradient drives charge carrier diffusion and produces a measurable PTE voltage [17, 18]. To establish the temperature gradient, half of each doped PBBT2T‐T film was covered with an opaque plastic plate, with the covered portion defined as the cold side. The measurement configuration is illustrated in Figure 3a. As shown in Figure 3c, under SWIR illumination of 10, 30, and 50 mW cm^−^^2^ for 30 s, the doped PBBT2T‐T films generated maximum output voltages of 108.1, 210.7, and 329.8 µV, respectively. Figure 3d shows a linear correlation between the output voltage and the induced temperature difference, from which the OPTE Seebeck coefficient (*S_OPTE_*) was determined to be 54.1 µV K^−1^. All values represent the mean of five independent devices. This value closely matches the Seebeck coefficient obtained from OTE measurements (*S_OTE_* = 54.6 µV K^−1^), confirming that the doped PBBT2T‐T efficiently converts incident SWIR light into electrical energy via the PTE effect. Furthermore, the output performance of the doped PBBT2T‐T was systematically evaluated under varying SWIR illumination intensities. Figure 3e shows that at an incident light intensity of 10 mW cm^−2^, the OPTE exhibited an open‐circuit voltage (*V_oc_*) of 108.0 µV, a short‐circuit current (*I_sc_*) of 0.66 µA, and an output power (*P_out_*) of 0.017 nW. When the light intensity increased to 30 mW cm^−2^, *V_oc_* and *I_sc_* rose to 210.8 µV and 1.65 µA, yielding a *P_out_* of 0.087 nW. At 50 mW cm^−2^, the OPTE achieved optimal performance with *V_oc_* = 329.6 µV, *I_sc_* = 2.67 µA, and maximum *P_out_* of 0.22 nW. These results indicate that both *V_oc_* and *I_sc_* increase approximately linearly with SWIR intensity (Figure S27), primarily due to the larger temperature gradient under higher photon flux, which enhances carrier diffusion and OPTE voltage generation. Long‐term operational stability was demonstrated by continuous illumination tests conducted at 50 mW cm^−2^ and 40% relative humidity, in which the output voltage remained nearly unchanged over 36 000 s (∼10 h) (Figure 3f).

Notably, PBBT2T‐T, due to its open‐shell diradicaloid structure, exhibits optical absorption extending deep into the SWIR region, far beyond the typical visible‐to‐NIR range (λ < 1000 nm) typically accessible to conventional organic photovoltaic (OPV) materials (Figure 3g). In OPVs, precise energy‐level alignment between donor and acceptor components is essential, as an excessively narrow bandgap in either component can hinder proper energy‐level matching and limit exciton dissociation and charge separation, thereby restricting the short‐circuit current density (*J*_sc_) and open‐circuit voltage (*V*_oc_) and ultimately constraining device performance [16]. Figure S2 schematically illustrates the OPV working principle and how narrow‐bandgap donors or acceptors affect the performance of photovoltaic devices. In contrast, OPTE systems can operate using a single active material combined with a small amount of molecular dopant, thereby bypassing the stringent energy level matching requirements inherent to donor‐acceptor heterojunction‐based devices. Consequently, doped PBBT2T‐T can directly harvest previously underutilized SWIR solar energy, which constitutes roughly one‐third of the solar spectrum, effectively complementing OPV absorption, and enhancing overall solar energy utilization. This capability highlights the strong potential of open‐shell OPTE materials for integration with advanced photovoltaic technologies to achieve broader solar‐spectrum utilization.

SWIR detection plays a crucial role in many scientific and technological applications, with broad uses including night vision, biomedical diagnostics, and optical communications. However, as the detection wavelength increases, the development of organic photodetectors (OPDs) becomes increasingly constrained, and achieving stable operation beyond 1500 nm remains particularly challenging (Figure S3). This limitation arises because organic donor or acceptor materials capable of absorbing in this spectral region generally possess extremely narrow bandgaps, which severely restricts energy‐level alignment within donor–acceptor heterojunctions, thereby reducing charge separation efficiency and photocurrent generation (Figure S2). To address these challenges, OPTE‐based detection serves as a promising alternative strategy that does not rely on exciton separation or strict energy‐level matching.

Based on this concept, doped PBBT2T‐T was evaluated as an organic photothermoelectric detector (OPTED) for detecting SWIR light with wavelengths exceeding 1500 nm. The experimental setup was identical to that shown in Figure 3a, except that the long‐pass filter was replaced with a cutoff wavelength of 1500 nm. As shown in Figure 4a, IR thermal images of doped PBBT2T‐T under different illumination intensities (auto‐scaled) are presented, and the corresponding time‐dependent temperature profiles are provided in Figure S28. Under SWIR illumination at intensities of 5, 10, and 15 mW cm^−2^ for 30 s, the film surface temperature increased by 0.8, 1.6, and 2.4 K, respectively, while the doped PBBT2T‐T simultaneously generated maximum output voltages of 43.3, 86.5, and 129.9 µV, as shown in Figure 4b. As presented in Figure 4c, the output voltage exhibits a linear dependence on the induced temperature difference, from which the OPTED Seebeck coefficient (*S_OPTED_*) was calculated to be 54.2 µV K^−1^. All reported values are averaged over five independent devices. This value is highly consistent with the Seebeck coefficient obtained from OTE measurements (*S_OTE_* = 54.6 µV K^−1^) as well as from the OPTE measurements (*S_OPTE_* = 54.1 µV K^−1^), confirming that the voltage response originates from a consistent intrinsic PTE mechanism.

**FIGURE 4:**
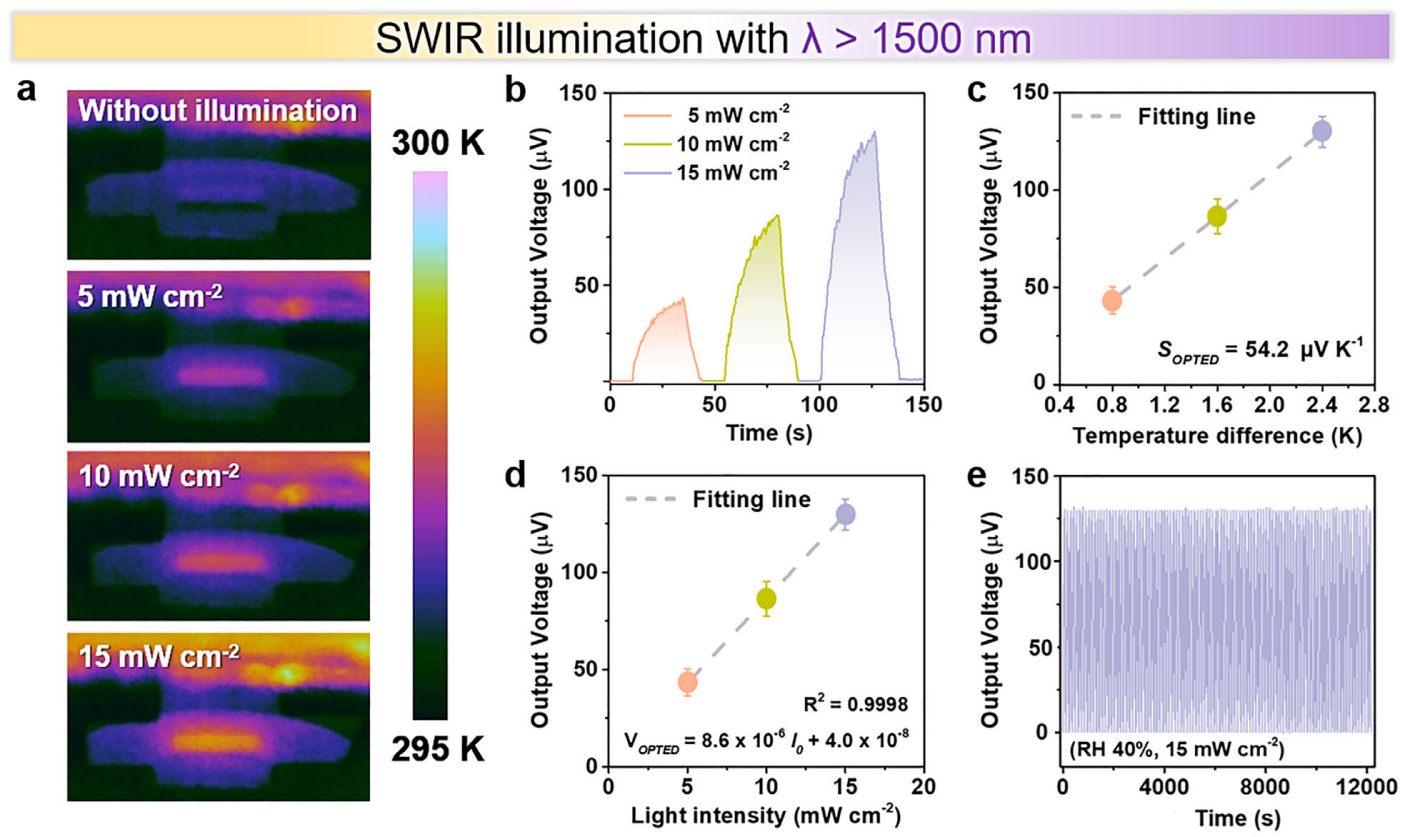
(a) IR images and (b) time‐dependent output voltage of the OPTED under different SWIR (λ > 1500 nm) intensities. (c) Linear correlation between output voltage and temperature difference for OPTED. (d) Linear correlation between output voltage and SWIR (λ > 1500 nm) intensity. (e) Photostability test: 100 light on/off cycles of the OPTED under 15 mW cm^−2^ SWIR (λ > 1500 nm) illumination at 40% relative humidity.

Speed is an important performance metric in photodetection. Under an illumination intensity of 15 mW cm^−2^, the OPTED device exhibits rise and fall times (*t_r_* and *t_f_*) of 13.5 s, corresponding to an estimated −3 dB bandwidth of approximately 0.026 Hz (Equation S6) [57, 58]. This relatively slow response is primarily governed by thermally driven processes that dominate the PTE response. Figure 4d illustrates the strong linear relationship between the OPTED output voltage and the incident illumination intensity, described by a linear fit of *V_OPTED_* = 8.6 × 10^−6^ *I_o_* + 4.0 × 10^−8^ with a correlation coefficient *R^2^* = 0.9998. Here, *V_OPTED_* corresponds to the OPTED voltage (µV) and *I_o_* represents the light intensity (mW cm^−2^). This high degree of linearity indicates that the doped PBBT2T‐T enables precise and quantitative SWIR light intensity detection, highlighting its suitability as a reliable OPTED material. The OPTED responsivity (*R*) was calculated using Equation (1) [58, 59]:

(1) $$R=\frac{V_{\mathrm{OPTED}}}{I_{0}A}$$

where *A* is the active area of the OPTED film. The responsivity of the doped PBBT2T‐T was determined to be 0.0063 V W^−^^1^. The Johnson voltage noise was calculated to be 4.8 × 10^−9^ V (Equation S7), from which the noise equivalent power (NEP) was determined as 3.0 × 10^−7^ W (Equation S8) based on the OPTED responsivity. The specific detectivity (D*) was then estimated using the Johnson‐noise‐limited equation [58, 59, 60, 61]:

(2) $$D^{*}=\frac{\sqrt{A}}{NEP}\;$$

The calculated D^*^ of 8.4 × 10^5^ Jones demonstrates that the doped PBBT2T‐T can effectively detect SWIR signals with wavelengths exceeding 1500 nm. The operational stability of the OPTD response was evaluated under repeated illumination. After 100 light on/off cycles at 15 mW cm^−2^ and 40% relative humidity, negligible degradation in OPTED voltage response was observed (Figure 4e), demonstrating good cycling stability.

These results indicate that an open‐shell conjugated polymer with intrinsic absorption beyond 1500 nm, combined with molecular doping, enables voltage‐based OPTED, allowing reliable detection of SWIR signals above 1500 nm without relying on donor‐acceptor heterojunctions or photocurrent generation, and providing the first demonstration that SWIR detection can be achieved via the OPTE effect. Although the performance of current OPTE‐based SWIR sensors remains limited, they show significant potential for further optimization and future applications. Compared with conventional current‐mode OPDs, voltage‐based OPTED devices support self‐powered operation, inherently avoids dark‐current limitations, offering a promising route for low‐noise SWIR sensing technologies.

## 3 Conclusions

In conclusion, PBBT2T‐T exhibits a pronounced open‐shell diradical character, enabling broad absorption from the visible to the SWIR region. Under SWIR illumination (>1000 nm, 50 mW cm^−2^), the F4TCNQ‐doped PBBT2T‐T OPTE delivered a *V_oc_* of 329.6 µV, an *I_sc_* of 2.67 µA, and a *P_out_* of 0.22 nW, while maintaining stability over 36 000 s of continuous illumination. Furthermore, the doped PBBT2T‐T enables OPTED in the challenging >1500 nm region, exhibiting a *R* of 0.0063 V W^−1^ and a D* of 8.4 × 10^5^ Jones, remaining stable over 100 on/off cycles. Beyond demonstrating SWIR‐responsive OPTE devices, these results highlight the critical role of open‐shell electronic structures in extending the absorption of conjugated polymers into the deep infrared while preserving effective charge generation through molecular doping. The synergistic combination of open‐shell polymer design and controlled doping therefore provides a viable strategy for overcoming the bandgap limitations typically encountered in organic optoelectronic systems. More broadly, this approach offers new opportunities for developing solution‐processable materials for SWIR energy harvesting and photodetection, potentially enabling lightweight, and flexible infrared sensing technologies.

## Author Contributions

**Guan‐Lin Chen**: conceptualization, methodology, software, data curation, formal analysis, validation, investigation, Writing – original draft, Writing – review and editing, visualization. **Shao‐Huan Hong**: conceptualization, validation. **Soonyong Lee**: conceptualization, methodology, data curation, formal analysis, investigation, writing – original draft. **Han Young Woo**: conceptualization, writing – review and editing, funding acquisition, methodology, supervision. **Jhih‐Min Lin**: resources. **Cheng‐Liang Liu**: conceptualization, writing – review and editing, supervision, methodology, funding acquisition.

## Funding

National Research Foundation of Korea (NRF) government grant (RS‐2025‐24683763); Taiwan National Science and Technology Council (NSTC), 2030 Cross‐Generation Young Scholars Program (114‐2628‐E‐002‐005); Academic Research‐Career Development Project (Sprout Research Projects), National Taiwan University (114L7817); Advanced Research Center For Green Materials Science and Technology, Featured Area Research Center Program of the Higher Education Sprout Project, Ministry of Education (114L9006).

## Conflicts of Interest

The authors declare no conflicts of interest.

## Supporting information




**Supporting File**: advs77195‐sup‐0001‐SuppMat.docx.

## Data Availability

The data that support the findings of this study are available from the corresponding author upon reasonable request.

## References

[1] O. S. Oliinyk , C. Ma , S. Pletnev , et al., “Deep‐Tissue SWIR Imaging Using Rationally Designed Small Red‐Shifted Near‐Infrared Fluorescent Protein,” Nature Methods 20, no. 1 (2023): 70–74, 10.1038/s41592-022-01683-0. PMC10725253 36456785

[2] H. Wu , Q. Shao , M. Liu , et al., “Metallic Fabry‐Pérot Cavity‐Enhanced "Pseudo‐Charge Transfer" Absorption for Efficient Narrowband Short‐Wave Infrared Photodetection,” Advanced Materials 37, no. 43 (2025): 09521, 10.1002/adma.202509521. 40810644

[3] F. Cao , L. Liu , and L. Li , “Short‐Wave Infrared Photodetector,” Materials Today 62 (2023): 327–349, 10.1016/j.mattod.2022.11.003.

[4] D. J. Naczynski , M. C. Tan , M. Zevon , et al., “Rare‐Earth‐Doped Biological Composites as in Vivo Shortwave Infrared Reporters,” Nature Communications 4, no. 1 (2013): 2199, 10.1038/ncomms3199. PMC3736359 23873342

[5] R. Li , Y. Zhang , S. Li , et al., “Shortwave Infrared Organic Optoelectronic Materials and Devices: Organic Photodetectors, Organic Solar Cells and Organic Light‐Emitting Diodes,” Advanced Functional Materials 36, no. 36 (2026): 32119, 10.1002/adfm.202532119.

[6] H. Tan , X. Wu , S. Hu , et al., “High‐Performance Organic Semiconductor Near‐Infrared and Shortwave‐Infrared Photodetectors: A Materials and Device Roadmap,” Chemical Science 16, no. 46 (2025): 21705–21744, 10.1039/D5SC06197K. PMC12598621 41221113

[7] Y. Wang , H. Wu , C. Rodà , et al., “Shortwave Infrared Light Detection and Ranging Using Silver Telluride Quantum Dots,” Advanced Materials 37, no. 21 (2025): 2500977, 10.1002/adma.202500977. PMC12107218 40159880

[8] F. P. García de Arquer , A. Armin , P. Meredith , and E. H. Sargent , “Solution‐Processed Semiconductors for Next‐Generation Photodetectors,” Nature Reviews Materials 2 (2017): 16100, 10.1038/natrevmats.2016.100.

[9] C. Fuentes‐Hernandez , W.‐F. Chou , T. M. Khan , et al., “Large‐Area Low‐Noise Flexible Organic Photodiodes for Detecting Faint Visible Light,” Science 370, no. 6517 (2020): 698–701, https://www.science.org/doi/10.1126/science.aba2624. 10.1126/science.aba2624 33154137

[10] X. Zhao , J. Wang , M. Liu , X. Ma , and F. Zhang , “Narrowband Organic Photodetectors: From Fundamentals to Prospects,” Advanced Optical Materials 12, no. 31 (2024): 2401087, 10.1002/adom.202401087.

[11] C. Wu , Z. Zhang , and T. Le , “Doped State Engineering in Conjugated Polymers,” Chemistry of Materials 38, no. 1 (2026): 3–19, 10.1021/acs.chemmater.5c02592.

[12] T. Cao , X.‐L. Shi , and Z.‐G. Chen , “Advances in the Design and Assembly of Flexible Thermoelectric Device,” Progress in Materials Science 131 (2023): 101003, 10.1016/j.pmatsci.2022.101003.

[13] Z. Ye , M. Zhang , J. Deng , L. Liang , C. Du , and G. Chen , “An Emerging Liquid‐Crystalline Conducting Polymer Thermoelectrics: Opportunities and Challenges,” Nano‐Micro Letters 18, no. 1 (2026): 82, 10.1007/s40820-025-01916-9. PMC12618801 41239068

[14] X. Hao , J. Wang , and H. Wang , “High Power Output Density Organic Thermoelectric Devices for Practical Applications in Waste Heat Harvesting,” Chemical Society Reviews 54, no. 4 (2025): 1957–1985, 10.1039/D4CS01045K. 39831346

[15] D. Wang , D. Zhu , and C.‐A. Di , “Engineering Molecular Assembly for High Performance Plastic Thermoelectrics,” Accounts of Chemical Research 59, no. 6 (2026): 932–944, 10.1021/acs.accounts.5c00867. 41789924

[16] A. Wadsworth , Z. Hamid , J. Kosco , N. Gasparini , and I. McCulloch , “The Bulk Heterojunction in Organic Photovoltaic, Photodetector, and Photocatalytic Applications,” Advanced Materials 32, no. 38 (2020): 2001763, 10.1002/adma.202001763. 32754970

[17] S. Lee , G.‐L. Chen , S. W. Oh , C.‐L. Liu , and H. Y. Woo , “Emerging Hot Issues in Organic Thermoelectrics,” ACS Applied Electronic Materials 7, no. 21 (2025): 9643–9659, 10.1021/acsaelm.5c01873.

[18] M. Dai , X. Zhang , and Q. J. Wang , “2D Materials for Photothermoelectric Detectors: Mechanisms, Materials, and Devices,” Advanced Functional Materials 34, no. 21 (2024): 2312872, 10.1002/adfm.202312872.

[19] X. Cui , Q. Ruan , X. Zhuo , et al., “Photothermal Nanomaterials: A Powerful Light‐to‐Heat Converter,” Chemical Reviews 123, no. 11 (2023): 6891–6952, 10.1021/acs.chemrev.3c00159. PMC10273250 37133878

[20] X. Zhang , D. Wang , L. Liu , et al., “Irregular Hierarchical‐Porous Polymer for High‐Performance Soft Thermoelectrics,” Science 391, no. 6789 (2026): 1063–1069, https://www.science.org/doi/10.1126/science.adx9237. 10.1126/science.adx9237 41785344

[21] S. Wang , G. Zuo , J. Kim , and H. Sirringhaus , “Progress of Conjugated Polymers as Emerging Thermoelectric Materials,” Progress in Polymer Science 129 (2022): 101548, 10.1016/j.progpolymsci.2022.101548.

[22] B. Dörling , I. E. Jacobs , I. Brunetti , et al., “Toward a Consensus Characterization Protocol for Organic Thermoelectrics,” Advanced Materials 38 (2026): 20430, 10.1002/adma.202520430. PMC12966978 41641891

[23] S. Zhou , X.‐L. Shi , L. Li , et al., “Advances and Outlooks for Carbon Nanotube‐Based Thermoelectric Materials and Devices,” Advanced Materials 37, no. 13 (2025): 2500947, 10.1002/adma.202500947. PMC11962713 39955649

[24] Y. Gao , Y. Liu , X. Li , et al., “A Stable Open‐Shell Conjugated Diradical Polymer With Ultra‐High Photothermal Conversion Efficiency for NIR‐II Photo‐Immunotherapy of Metastatic Tumor,” Nano‐Micro Letters 16, no. 1 (2024): 21, 10.1007/s40820-023-01219-x. PMC10660627 37982963

[25] Q. Wei , J. Huang , Q. Meng , Z. Zhang , S. Gu , and Y. Li , “Open‐Shell Poly(3,4‐dioxythiophene) Radical for Highly Efficient Photothermal Conversion,” Advanced Science 11, no. 41 (2024): 2406800, 10.1002/advs.202406800. PMC11538641 39234816

[26] L. Tian , J. Zhang , M. Shang , et al., “Open‐Shell Polymers Reaching High Photocatalysis and Photothermal Effect,” Chemical Engineering Journal 509 (2025): 161349, 10.1016/j.cej.2025.161349.

[27] F. Jiang and M. Wang , “Room‐Temperature Ferromagnetism and Near‐Infrared Luminescence Enabled by Interchain‐Aggregation Control of Open‐Shell Donor–Acceptor Alternating Copolymers,” Macromolecules 58, no. 3 (2025): 1409–1424, 10.1021/acs.macromol.4c02787.

[28] T. L. D. Tam , G. Wu , S. W. Chien , S. F. V. Lim , S.‐W. Yang , and J. Xu , “High Spin Pro‐Quinoid Benzo[1,2‐c;4,5‐c′]Bisthiadiazole Conjugated Polymers for High‐Performance Solution‐Processable Polymer Thermoelectrics,” ACS Materials Letters 2 (2020): 147–152, 10.1021/acsmaterialslett.9b00483.

[29] J. Casado , R. P. Ortiz , and J. T. López Navarrete , “Quinoidal Oligothiophenes: New Properties behind an Unconventional Electronic Structure,” Chemical Society Reviews 41, no. 17 (2012): 5672–5686, 10.1039/C2CS35079C. 22806576

[30] T. Takahashi , K. Matsuoka , K. Takimiya , T. Otsubo , and Y. Aso , “Extensive Quinoidal Oligothiophenes With Dicyanomethylene Groups at Terminal Positions as Highly Amphoteric Redox Molecules,” Journal of the American Chemical Society 127, no. 25 (2005): 8928–8929, 10.1021/ja051840m. 15969551

[31] L. Huang , N. Eedugurala , A. Benasco , et al., “Open‐Shell Donor–Acceptor Conjugated Polymers With High Electrical Conductivity,” Advanced Functional Materials 30, no. 24 (2020): 1909805, 10.1002/adfm.201909805.

[32] M. E. Steelman , D. J. Adams , K. S. Mayer , et al., “Magnetic Ordering in a High‐Spin Donor–Acceptor Conjugated Polymer,” Advanced Materials 34 (2022): 2206161, 10.1002/adma.202206161. 36114614

[33] Y. Li , L. Li , Y. Wu , and Y. Li , “A Review on the Origin of SyntheticMetal Radical: Singlet Open‐Shell Radical Ground State?,” The Journal of Physical Chemistry C 121, no. 15 (2017): 8579–8588, 10.1021/acs.jpcc.6b12936.

[34] Z. Chen , W. Li , Y. Zhang , et al., “Aggregation‐Induced Radical of Donor–Acceptor Organic Semiconductors,” The Journal of Physical Chemistry Letters 12, no. 40 (2021): 9783–9790, 10.1021/acs.jpclett.1c02463. 34596405

[35] X. Ji and L. Fang , “Quinoidal Conjugated Polymers With Open‐Shell Character,” Polymer Chemistry 12, no. 10 (2021): 1347–1361, 10.1039/D0PY01298J.

[36] Y. Joo , L. Huang , N. Eedugurala , et al., “Thermoelectric Performance of an Open‐Shell Donor–Acceptor Conjugated Polymer Doped With a Radical‐Containing Small Molecule,” Macromolecules 51, no. 10 (2018): 3886–3894, 10.1021/acs.macromol.8b00582.

[37] Y. Kim , Y.‐J. Kim , Y.‐A. Kim , et al., “Open‐Shell and Closed‐Shell Quinoid–Aromatic Conjugated Polymers: Unusual Spin Magnetic and High Charge Transport Properties,” ACS Applied Materials & Interfaces 13, no. 2 (2021): 2887–2898, 10.1021/acsami.0c15893. 33404212

[38] R. Wu , Y. Wei , X. Dai , et al., “Thermoelectric Performance in Triplet‐Ground‐State Polymer Intrinsically Boosted by Enhanced Proquinoidal Characteristic,” Angewandte Chemie International Edition 64 (2025): 202413061, 10.1002/anie.202413061. 39140438

[39] J.‐F. Ding , G.‐L. Chen , P.‐H. Liu , et al., “Enhancing the Thermoelectric Performance of Donor–Acceptor Conjugated Polymers Through Dopant Miscibility: A Comparative Study of Fluorinated Substituents and Side‐Chain Lengths,” Journal of Materials Chemistry A 12, no. 16 (2024): 9806–9816, 10.1039/D4TA00032C.

[40] Y. H. Shih , G. L. Chen , P. H. Liu , et al., “Revealing the Effect of Branched Side Chain Length on Polymer Aggregation and Paracrystallinity for Improved Mobility–Stretchability Properties,” ACS Applied Electronic Materials 6, no. 3 (2024): 1797–1808, 10.1021/acsaelm.3c01719.

[41] S. Sharma , M. Desu , G. L. Chen , et al., “Enhancing Optoelectronic Anisotropy in Highly Oriented Thin Films by Fluorine Substitution in Novel Semiconducting Polymers,” ACS Applied Materials & Interfaces 16, no. 38 (2024): 51229–51240, https://pubs.acs.org/doi/10.1021/acsami.4c08566. 10.1021/acsami.4c08566 PMC11440466 39285684

[42] J. Tirado‐Rives and W. L. Jorgensen , “Performance of B3LYP Density Functional Methods for a Large Set of Organic Molecules,” Journal of Chemical Theory and Computation 4, no. 2 (2008): 297–306, 10.1021/ct700248k. 26620661

[43] J.‐F. Ding , K. Yamanaka , S.‐H. Hong , et al., “Controlling the Thermoelectric Performance of Doped Naphthobisthiadiazole‐Based Donor–Acceptor Conjugated Polymers Through Backbone Engineering,” Advanced Science 11, no. 48 (2024): 2410046, 10.1002/advs.202410046. PMC11672273 39488764

[44] Q.‐B. Zheng , Y.‐C. Lin , Y.‐T. Lin , et al., “Investigating the Stretchability of Doped Poly(3‐hexylthiophene)‐Block‐Poly(butylacrylate) Conjugated Block Copolymer Thermoelectric Thin Films,” Chemical Engineering Journal 472 (2023): 145121, 10.1016/j.cej.2023.145121.

[45] K. Feng , W. Yang , S. Y. Jeong , et al., “Cyano‐Functionalized Fused Bithiophene Imide Dimer‐Based n‐Type Polymers for High‐Performance Organic Thermoelectrics,” Advanced Materials 35, no. 31 (2023): 2210847, 10.1002/adma.202210847. 37120703

[46] L. Tu , J. Wang , Z. Wu , et al., “Cyano‐Functionalized Pyrazine: A Structurally Simple and Easily Accessible Electron‐Deficient Building Block for n‐Type Organic Thermoelectric Polymers,” Angewandte Chemie International Edition 136 (2024): 202319658, 10.1002/ange.202319658. 38265195

[47] Y. Li , W. Wu , Y. Wang , et al., “Multi‐Selenophene Incorporated Thiazole Imide‐Based n‐Type Polymers for High‐Performance Organic Thermoelectrics,” Angewandte Chemie International Edition 136 (2024): 202316214, 10.1002/ange.202316214. 37996990

[48] S. Gámez‐Valenzuela , J. Li , S. Ma , et al., “High‐Performance n‐Type Organic Thermoelectrics With Exceptional Conductivity by Polymer‐Dopant Matching,” Angewandte Chemie International Edition 136 (2024): 202408537, 10.1002/ange.202408537. 38973771

[49] J. E. Cochran , M. J. N. Junk , A. M. Glaudell , et al., “Molecular Interactions and Ordering in Electrically Doped Polymers: Blends of PBTTT and F4TCNQ,” Macromolecules 47, no. 19 (2014): 6836–6846, 10.1021/ma501547h.

[50] J. Min , D. Kim , S. G. Han , et al., “Position‐Induced Efficient Doping for Highly Doped Organic Thermoelectric Materials,” Advanced Electronic Materials 8, no. 3 (2022): 2101142, 10.1002/aelm.202101142.

[51] T. H. Kim , S. Y. Jeong , S. Oh , et al., “Control of Chemical Doping‐Mediated Space Charge for Energetic Band‐Switching Modulation in Low‐Noise Shortwave‐Infrared Organic Photodetector,” Advanced Materials 37, no. 30 (2025): 2500126, 10.1002/adma.202500126. 40346779

[52] A. R. Umar , A. L. Dorris , and C. Grieco , “Photoexcited Polaron Relaxation as a Structurally Sensitive Reporter of Charge Trapping in a Conducting Polymer,” Advanced Functional Materials 34, no. 46 (2024): 2407181, 10.1002/adfm.202407181.

[53] K. E. Watts , B. Neelamraju , E. L. Ratcliff , and J. E. Pemberton , “Stability of Charge Transfer States in F4TCNQ‐Doped P3HT,” Chemistry of Materials 31 (2019): 6986–6994, 10.1021/acs.chemmater.9b01549. 30450910

[54] S. E. Yoon , J. Park , J. E. Kwon , et al., “Improvement of Electrical Conductivity in Conjugated Polymers Through Cascade Doping With Small‐Molecular Dopants,” Advanced Materials 32, no. 49 (2020): 2005129, 10.1002/adma.202005129. 33135210

[55] C. G. Tang , K. Hou , and W. L. Leong , “The Quest for Air Stability in Organic Semiconductors,” Chemistry of Materials 36, no. 1 (2024): 28–53, 10.1021/acs.chemmater.3c02093.

[56] M. Jha , J. Mogollon Santiana , A. A. Jacob , et al., “Stability Study of Molecularly Doped Semiconducting Polymers,” The Journal of Physical Chemistry C 128, no. 3 (2024): 1258–1266, 10.1021/acs.jpcc.3c06044.

[57] Y.‐H. Deng , D. Van , and T. Z. Hens , “Understanding and Equivalence of Response Time Measurements in Photodetectors,” ACS Photonics 13, no. 7 (2026): 1752–1756, 10.1021/acsphotonics.6c00438.

[58] S. Wredh , M. Dai , K. Hamada , et al., “Sb_2_Te_3_–Bi_2_Te_3_ Direct Photo–Thermoelectric Mid‐Infrared Detection,” Advanced Optical Materials 12, no. 31 (2024): 2401450, 10.1002/adom.202401450.

[59] X. He , F. Leonard , and J. Kono , “Uncooled Carbon Nanotube Photodetectors,” Advanced Optical Materials 3, no. 8 (2015): 989–1011, 10.1002/adom.201500237.

[60] J. Wang , Z. Xie , J. A. Liu , and J. T. Yeow , “Design of Room‐Temperature Infrared Photothermoelectric Detectors Based on CNT/PEDOT:PSS Composites,” Journal of Materials Chemistry C 10, no. 40 (2022): 15105–15113, 10.1039/D2TC03159K.

[61] E. Kaminski , N. Suleymanov , B. Minkovich , et al., “Waveguide Integrated Self‐Powered MoS_2_ Photodetectors in the Shortwave Infrared Wavelengths,” ACS Photonics 12, no. 11 (2025): 6397–6405, 10.1021/acsphotonics.5c01893. PMC12636074 41283014

