# Supplementary material for: Open‐Shell Conjugated Polymers Enable Photothermoelectric Conversion and Detection of Short‐Wave Infrared Light
Source: Adv Sci (Weinh). 2026 Aug 19:e77195. Online ahead of print. doi: 10.1002/advs.77195 (PMC13487860; doi:10.1002/advs.77195)
Supplement: Supplementary file 1 [file ADVS-9999-e77195-s001.docx]

Supporting Information

**Open-Shell Conjugated Polymers Enable Photothermoelectric Conversion and Detection of Short-Wave Infrared Light**

*Guan-Lin Chen, Soonyong Lee, Shao-Huan Hong, Jhih‐Min Lin, Han Young Woo* and Cheng-Liang Liu**

G.-L. Chen, S.-H. Hong, C.-L. Liu

Department of Materials Science and Engineering, National Taiwan University, Taipei 10617, Taiwan

E-mail: [liucl@ntu.edu.tw](mailto:liucl@ntu.edu.tw)

S. Lee, H. Y. Woo

Department of Chemistry, Korea University, Seoul 02841, Republic of Korea

E-mail: [hywoo@korea.ac.kr](mailto:hywoo@korea.ac.kr)

J.‐M. Lin

National Synchrotron Radiation Research Center, Hsinchu 30076, Taiwan

C.-L. Liu

Advanced Research Center for Green Materials Science and Technology, National Taiwan University, Taipei 10617, Taiwan

Keywords: open-shell structure, short-wave infrared, photothermoelectric, detector

**Experimental section**

**Materials**

All reagents and solvents were purchased from commercial suppliers and used as received unless otherwise noted. 4,7-bis(5-bromo-4-(2-octyldodecyl)thiophen-2-yl)benzo[c][1,2,5]thiadiazole, and 4,8-bis(5-bromo-4-(2-octyldodecyl)thiophen-2-yl)benzo[1,2-c;4,5-c′]bis[1,2,5]thiadiazole were synthesized according to previously reported procedures. ^[1, 2]^ 2,5-Bis(trimethylstannyl)thiophene was purchased from Sigma-Aldrich and used as received.

**Polymer Synthesis**

**Scheme S1.** Synthetic routes of PBT2T-T and PBBT2T-T.

**PBT2T-T**

In a 10 mL microwave reaction vial, 4,7-bis(5-bromo-4-(2-octyldodecyl)thiophen-2-yl)benzo[c][1,2,5]thiadiazole (122.31 mg, 0.12 mmol) and 2,5-bis(trimethylstannyl)thiophene (49.17 mg, 0.12 mmol) were combined with Pd(PPh_3_)_4_ (11.55 mg, 0.01 mmol) as the catalyst. The reaction mixture was dissolved in 4.7 mL of anhydrous chlorobenzene, giving an overall monomer concentration of 0.025 M. The vial was sealed with a PTFE cap and subjected to microwave irradiation at 150 °C for 3.5 h. After completion of the reaction, the mixture was slowly poured into methanol to precipitate the polymer. The resulting solid was collected by filtration through a fritted funnel. The crude polymer (PBT2T-T) was further purified by Soxhlet extraction using methanol, acetone, hexane, dichloromethane (DCM), and chloroform (CHCl₃) sequentially. The final product was obtained as a dark blue solid (105.1 mg, 61.3% yield). ^1^H NMR (600 MHz, CDCl_3_): δ (ppm) 8.05 (br, 2H), 7.91 (br, 2H), 2.79 (br, 4H), 1.82 (br, 2H), 1.34-1.22 0.85 (br, 76H), 0.88-0.85 (t, 12H).

**PBBT2T-T**

In a 5 mL microwave reaction vial, 4,8-bis(5-bromo-4-(2-octyldodecyl)thiophen-2-yl)benzo[1,2-c;4,5-c′]bis[1,2,5]thiadiazole (150 mg, 0.14 mmol) and 2,5-bis(trimethylstannyl)thiophene (57 mg, 0.14 mmol) were combined with Pd_2_(dba)_3_ (6.35 mg, 0.0069 mmol) and P(o-tol)_3_ (6.35 mg, 0.021 mmol) as the catalytic system. The reagents were dissolved in 3 mL of anhydrous xylene. The vial was sealed with a PTFE cap and heated at 150 °C for 48 h in an oil bath. After completion of the reaction, the mixture was slowly added dropwise into methanol to precipitate the conjugated polymer. The resulting solid was collected by filtration through a fritted funnel. The crude polymer (PBBT2T-T) was further purified by Soxhlet extraction using methanol, acetone, hexane, and chloroform sequentially. The final product was obtained as a dark blue solid (67 mg, 32.4% yield). ^1^H NMR (600 MHz, CDCl_3_): *δ* (ppm) 9.0 (br, 2H), 7.5 (br, 2H), 4.88 (br, 4H), 2.90 (br, 2H), 2.14-0.50 (br, 76H).

**Fabrication of Doped Conjugated Polymer Thin Films**

Polymer thin films were fabricated on glass substrates (15 mm × 15 mm). Prior to deposition, the substrates were sequentially cleaned by sonication in isopropanol, dried under a nitrogen stream, and treated with oxygen plasma for 2 min. Solutions of PBT2T-T and PBBT2T-T (10 mg mL^-1^ in chloroform) were spin-coated onto the cleaned substrates at 2000 rpm for 30 s, followed by thermal annealing at 100 °C for 15 min. Molecular doping was performed via a vapor-phase doping process. F4TCNQ (sublimation temperature ≈ 90 °C) was thermally sublimed at 230 °C under ambient pressure in a nitrogen-filled glovebox. ^[3]^ Approximately 2 mg of F4TCNQ was placed in a glass dish (3 cm in diameter and 1 cm in height) and used as the dopant source. Polymer-coated substrates were positioned ~1 cm above the dopant source to enable controlled vapor infiltration. This setup ensured a highly reproducible doping environment for all samples. A schematic illustration of the vapor-doping configuration is shown in **Figure S13**.

**OTE Measurements**

For thermoelectric characterization, silver paste was applied to both ends of the doped polymer films to serve as electrical contacts. Their thermoelectric properties, including electrical conductivity and Seebeck coefficient, were measured using a commercial ZEM-3 system (ADVANCE RIKO). All measurements were conducted at a stable ambient temperature of 30 °C, with temperature gradients of 10, 20, and 30 °C applied across the samples. The distance between the thermoelectric probes was maintained at approximately 4 mm to ensure measurement accuracy and reproducibility.

**SWIR OPTE and OPTED Measurements**

The hot side of the OPTE and OPTED devices was generated using a 300 W xenon lamp (Yamashita Denso YSS-80S, AAA grade) equipped with long-pass filters of >1000 nm (84-766, ZOLIX) and >1500 nm (FELH1500, Edmund Scientific Int’l Dept.), respectively, while a custom-built light shield served as the cold side of devices. A schematic illustration of the device setup is provided in **Figure S22**. This configuration enabled control over the incident light intensity to establish different temperature gradients across the devices. The short-wave infrared (SWIR) irradiance was quantitatively measured using a thermal detector (10A-BB-16-V2, Ophir Optronics Solutions Ltd.) coupled with a handheld optical power and energy meter (1919-R, Newport Corporation). The temperature evolution of the doped polymer films under SWIR illumination was monitored using a thermal infrared camera (FLIR A50). Under ambient conditions, a custom-built measurement system based on a Keithley 2400 sourcemeter was used to evaluate the electrical power generation and to acquire the voltage–time response, as well as current–voltage (*I–V*) and current–power (*I–P*) characteristics.

**Instrumentation**

^1^H NMR spectra were recorded on a Bruker 600 MHz spectrometer using CDCl_3_ as the solvent. Differential scanning calorimetry (DSC) measurements were performed on a PerkinElmer DSC 8000 with heating and cooling cycles between 50 and 250 °C at a rate of 10 °C min^-1^ under a nitrogen atmosphere. Thermogravimetric analysis (TGA) was conducted on a HITACHI STA 7200 under continuous nitrogen flow, with samples heated from 50 to 700 °C at 10 °C min^-1^. UV–vis–NIR absorption spectra were collected using a Hitachi U-4100 spectrophotometer. Atomic force microscopy (AFM) images of thin films were acquired in tapping mode under ambient conditions using a Hitachi AFM5100N equipped with SI-DF3PS tips (resonant frequency: 70 kHz; force constant: 2.0 N m^-1^). Grazing-incidence wide-angle X-ray scattering (GIWAXS) measurements were carried out at the TPS 25A beamline of the National Synchrotron Radiation Research Center (NSRRC, Taiwan). X-ray photoelectron spectroscopy (XPS) was performed on a PHI 5000 VersaProbe III (ULVAC-PHI, Inc.) under ultra-high vacuum (6.7 × 10^-7^ Pa) using He I radiation (21.22 eV) and monochromatic Al Kα X-rays (1486.6 eV) as excitation sources. Photoelectron spectroscopy in air (PESA) measurements were conducted using a Riken Keiki Surface Analyzer (AC-2S Pro Alpha DC) to determine the HOMO energy levels. Film thicknesses were obtained using an Alpha-Step® D300 surface profiler (KLA-Tencor). Carrier concentration and mobility of doped films were measured by a Hall effect system (HMS-3000, Ecopia). Electron paramagnetic resonance (EPR) spectra were acquired at room temperature in the X-band using a Bruker ELEXSYS-E580 spectrometer. Polymer molecular weights were determined by gel permeation chromatography (GPC) at 40 °C using tetrahydrofuran as the eluent at a flow rate of 1.0 mL min^-1^, calibrated against polystyrene standards.

**Table S1.** Summary of OPDs and performances based on previous beyond 850 nm reported in the literature.

| Photosensitive  materials | λ_onset_ of abs  (nm) | Laser excitation wavelength  (nm) | *R*  (A W^-1^) | *D*_sh_*  (Jones) | Ref. |
| --- | --- | --- | --- | --- | --- |
| PBDB-T:DO-4F | 1000 | 850 | 0.50 | 3.05 x 10^13^ | [4] |
| PTB7-Th:PCBM:O6T-4F | 1050 | 850 | 0.52 | 1.10 x 10^14^ | [5] |
| PBDT-TT:DPPSe-4Cl:Y6 | ~1060 | 950 | 0.18 | 1.0 x 10^13^ | [6] |
| PCE10:FB-C6 | 1150 | 970 | 0.29 | 2.39 x 10^12^ | [7] |
| PTB7-Th:  COTIC-4F:PC_71_BM | ~1140 | 940 | 0.53 | 3.4 x 10^13^ | [8] |
| PTB7-Th:YOR1 | 1000 | 950 | 0.141 | 1.98 x 10^13^ | [9] |
| PBDB-T: FM2 | 1040 | 880 | 0.455 | 1.01 x 10^13^ | [10] |
| PM6:ABTPV-S | ~1000 | 860 | 0.39/0.41 | 3.43 x 10^12^ | [11] |
| PM6:PDTTYM | 1020 | 900 | 0.48 | 1.31 × 10^13^ | [12] |
| PCE10:BTPV-4F-eC9 | 1050 | 900 | 0.56 | 4.6 × 10^13^ | [13] |
| PM6:CT-Cl | 1042 | 950 | 0.40 | 8.1 x 10^12^ | [14] |
| PCE10:YZ1 | 1050 | 1000 | 0.27 | 9.24 x 10^13^ | [15] |
| PTB7-Th:tSPT-4F | 1100 | 1010 | 0.40 | 1.25 x 10^13^ | [16] |
| PTB7-Th  :TTD(DTC-2FIC)_2_ | ~1350 | 1100 | 0.095 | 1.1 x 10^13^ | [17] |
| PTB7-Th:COT-Oct | ~1110 | 1000 | 0.24 | 1.49 x 10^12^ | [18] |
| PTTPQx:PC_61_BM | >800 | 1448 | - | 1.82 × 10^10^ | [19] |
| PTB7-Th:CS-1 | 1550 | 1300 | 0.015 | 2.96 × 10^10^ | [20] |
| BTT-DTPn-2F | 1320 | 1200 | 0.09 | 7.19 × 10^11^ | [21] |
| PM6:FeCl_3_ | >1500 | 1490 | 70 | 3.21 × 10^10^ | [22] |
| N2200/PPhTQ:COTIC-4F | 1600 | 1600 | 0.018 | 4.19 × 10^10^ | [23] |
| P3HT:F4TCNQ | >1600 | 1800 | 1120 | 1.11 × 10^11^ | [24] |
|  |  |  |  |  |  |

**Table S2.** Molecular weight, optical properties, and thermal properties of PBT2T-T and PBBT2T-T.

| Samples | *M_w_* (kDa)^a^ | PDI^a^ | HOMO (eV) ^b^ | *T_d, 95%_* (^o^C)^c^ | *T_m_* (^o^C) | *T_c_* (^o^C) |
| --- | --- | --- | --- | --- | --- | --- |
| PBT2T-T | 23.4 | 1.54 | -4.91 | 438 | 114 | 87 |
| PBBT2T-T | 11.4 | 2.06 | -4.69 | 373 | 297 | 246 |

^a^ Molecular weight was determined by using polystyrene standards and tetrahydrofuran (THF) as the eluent at 40 ^o^C.

^b^ HOMO energy levels were determined using PESA.

^c^ The temperature corresponding to 5% weight loss.

**Table S3.** Crystallographic parameters of pristine and doped films derived from 2D-GIWAXS analysis.

| Samples | Lamellar (100) spacing  [Å] | FWHM (100)  [Å^−1^] | *L_c_* (100)  [Å] | π - π (010) spacing  [Å] | FWHM (010)  [Å^−1^] | *L_c_*  (010)  [Å] |
| --- | --- | --- | --- | --- | --- | --- |
| PBT2T-T | 22.1  (OOP) | 0.02296  (OOP) | 273.6  (OOP) | - | - | - |
| Doped  PBT2T-T | 22.1  (OOP) | 0.02582  (OOP) | 243.3  (OOP) | - | - | - |
| PBBT2T-T | 23.2  (IP) | 0.03912  (IP) | 160.6  (IP) | 3.4  (OOP) | 0.17775  (OOP) | 35.3  (OOP) |
| Doped PBBT2T-T | 24.3  (IP) | 0.04643  (IP) | 135.32  (IP) | 3.4  (OOP) | 0.19258  (OOP) | 32.6  (OOP) |

**Absorption coefficient**

α(*υ*) is the absorption coefficient defined by the Beer-Lambert’s law (**Equation S1**). ^[25]^

$\alpha(\upsilon)= \frac{2.303 \times A_{max}}{d}$	(S1)

where *d* and *A_max_* are the film thickness and maximum absorbance, respectively.

**Activation energy**

The charge transport physics of the doped polymers, activation energies (*E_a_*) were extracted by measuring the temperature-dependent conductivity and fitting the results using the Arrhenius relation (**Equation S2**). ^[26]^

$\sigma=\sigma_{max}\exp\left( \frac{-E_{a}}{k_{B}T} \right)$	(S2)

where *σ_max_* represents the maximum conductivity at 323 K, *k_B_* is Boltzmann’s constant, and *T* is the absolute temperature.

**Calculation of doping efficiency**

The doping efficiency (*η_d_*) was determined from the N 1s XPS spectrum using **Equation S3**, defined as the ratio of the integrated area of the neutral peak (N^0^) to the total integrated area of the neutral (N⁰) and ionized (N^-1^) peaks: ^[27]^

$\eta_{d}= \frac{A^{-1}}{A^{0}+ A^{-1}}$	(S3)

where *A^0^* and *A^-1^* are the areas of the N^0^ and N^-1^ peaks, respectively.

**Organic thermoelectric (OTE)**

The Seebeck coefficient (*S_OTE_*) is fundamentally governed by the carrier concentration (*n*), as described by **Equation S4**: ^[28]^

$S_{OTE} = \frac{8\pi^{2}k_{B}^{2}T}{3eh^{2}}m\left( \frac{\pi}{3n} \right)^{\frac{2}{3}}$	(S4)

where *e* is the elementary charge, and *m* is the electron effective mass of charge carriers. Consequently, an inherent trade-off exists between the *S_OTE_* and *σ* due to their opposing dependence on *n*.

The OTE performance of the doped polymer films was assessed by measuring the *S_OTE_* and σ, from which the power factor (*PF*) was determined. (**Equation S5**). ^[29]^

$PF=S_{OTE}^{2}\sigma$	(S5)

**Response Bandwidth (*f_3dB_*)**

The response bandwidth is defined as the −3 dB cutoff frequency, at which the responsivity of the photodetector decreases to 70.7% of its low-frequency value. For a first-order system, the −3 dB bandwidth can be estimated from the 10–90% rise time (*t_r_*) using **Equation S6**: ^[30, 31]^

$f_{3dB}= \frac{0.35}{t_{r}}$	(S6)

**Noise equivalent power (NEP)**

The Johnson noise floor was estimated by assuming that the electrical resistance (*r*) of the doped PBBT2T-T channel is the primary dissipative element responsible for thermal fluctuations. The corresponding Johnson voltage noise is given by **Equation S7**: ^[31–34]^

$\upsilon_{n, Johnson}= \sqrt{4K_{B}Tr}$	(S7)

where *k_B_* is the Boltzmann constant, *T* is the absolute temperature.

Subsequently, the noise equivalent power (NEP) was calculated using **Equation S8**: ^[31-34]^

$NEP = \frac{\upsilon_{n}}{R}$	(S8)

where *R* is the OPTED responsivity.

**Figure S1.** The AM1.5G solar spectrum and the corresponding distribution of solar energy across various spectral regions.

**Figure S2.** a) Working principle of OPVs and OPDs. b) Impact of a narrow-bandgap donor on device performance. c) Impact of a narrow-bandgap acceptor on device performance.

**Figure S3.** Summary of the specific detectivities of OPDs operating in the NIR and SWIR regions, with the corresponding literature data listed in Table S1.

**Figure S4.** ^1^H NMR spectra of M1.

**Figure S5.** ^1^H NMR spectra of M2.

**Figure S6.** ^1^H NMR spectra of PBT2T-T.

**Figure S7.** ^1^H NMR spectra of PBBT2T-T.

**Figure S8.** a) TGA and b) DSC curves of PBT2T-T and PBBT2T-T.

**Figure 9.** a) UV–vis–NIR absorption spectra of PBT2T-T and PBBT2T-T thin films with different thicknesses. b) Linear fits of the maximum absorbance versus film thickness for PBT2T-T and PBBT2T-T thin films.

**Figure S10.** PESA spectra of PBT2T-T and PBBT2T-T films.

**Figure S11.** DFT-optimized frontier molecular orbitals of a) PBT2T-T and b) PBBT2T-T.

**Figure S12.** Molecular electrostatic potential (ESP) maps of a) PBT2T-T and b) PBBT2T-T.

**Figure S13.** Schematic illustration of vapor doping.

**Figure S14.** Hall effect characterization of the doped films: a) carrier concentration and b) carrier mobility.

**Figure S15.** Room-temperature EPR spectra of doped PBT2T-T and PBBT2T-T.

**Figure S16.** PESA spectra of a) pristine and doped PBT2T-T films and b) pristine and doped PBBT2T-T films.

**Figure S17.** Arrhenius plots of the electrical conductivity of doped films.

**Figure S18.** XPS spectra: S 2p of a) pristine and doped PBT2T-T and b) pristine and doped PBBT2T-T films.

**Figure S19.** XPS spectra: N 1s of a) doped PBT2T-T and b) doped PBBT2T-T films.

**Figure S20.** AFM height images and root-mean-square (RMS) surface roughness of pristine (top) and doped (bottom) films.

**Figure S21.** In-plane and out-of-plane grazing-incidence wide-angle X-ray scattering (GIWAXS) line-cut profiles of pristine and doped films: a) PBT2T-T and b) PBBT2T-T.

**Figure S22.** Photographs of the SWIR OPTE and OPTED measurement setup from the a,b) side views and c) top view.

**Figure S23**. Time-dependent temperature of pristine films under 50 mW cm^-2^ SWIR illumination.

**Figure S24**. Time-dependent temperature of doped PBBT2T-T films under different SWIR (λ > 1000 nm) illumination intensities.

**Figure S25.** OTE performance of doped PBBT2T-T films.

**Figure S26.** a) Air stability of doped PBBT2T-T OTEs at 25 °C and 40% relative humidity. b) Time-dependent UV–vis–NIR absorption spectra of doped PBBT2T-T films at 25 °C and 40% relative humidity.

**Figure S27.** Linear relationship between SWIR light intensity and both a) output voltage and b) current for OPTE devices.

**Figure S28.** Time-dependent temperature of doped PBBT2T-T films under different SWIR (λ > 1500 nm) illumination intensities.

**References**

[1] J.-F. Ding, G.-L. Chen, P.-H. Liu, et. al., “Enhancing the Thermoelectric Performance of Donor–Acceptor Conjugated Polymers Through Dopant Miscibility: a Comparative Study of Fluorinated Substituents and Side-Chain Lengths,” *Journal of Materials Chemistry A* 12 (2024): 9806–9816, <https://doi.org/10.1039/D4TA00032C>.

[2] R. Wu, Y. Wei, X. Dai, et. al., “Thermoelectric Performance in Triplet-Ground-State Polymer Intrinsically Boosted by Enhanced Proquinoidal Characteristic,” *Angewandte Chemie International Edition* 64 (2025): e202413061, <https://doi.org/10.1002/anie.202413061>.

[3] P. Kim, E. Y. Kim, S. G. Han, M. Jeong, K. Cho, and J. K. Kim, “High Vertical Hole Mobility through Minimization of Water-Induced Traps by F4TCNQ Doping in Conjugated Amphiphilic Diblock Copolymers,” *Macromolecules* 57 (2024): 4986−4992, <https://doi.org/10.1021/acs.macromol.4c00640>.

[4] Y. Xu, T. Zhang, H. Yao, J. Wang, P. Bi, and J. Hou, “Design of Ultranarrow-Bandgap Acceptors for Efficient Organic Photovoltaic Cells and Highly Sensitive Organic Photodetectors,” *Journal of Energy Chemistry* 72 (2022): 388–394, <https://doi.org/10.1016/j.jechem.2022.05.038>.

[5] C. Shan, T. Liu, J. Zhou, et. al., “High-Performance Organic Solar Cell and Self-power Photodetector with Chemically Robust, Near-Infrared Acceptor Enabled by Strengthening Interfacial Contact and Compositional Modulation,” *Chemical Engineering Journal* 471 (2023): 144451, <https://doi.org/10.1016/j.cej.2023.144451>.

[6]	Y. Wang, M. Yang, B. Yin, et. al., “An A–D–A′–D–A-Type Narrow Bandgap Electron Acceptor Based on Selenophene-Flanked Diketopyrrolopyrrole for Sensitive Near-Infrared Photodetection,” *ACS Applied Materials & Interfaces* 16 (2024): 66846–66856, <https://doi.org/10.1021/acsami.3c15365>.

[7]	Y. Wang, J. Wang, J. Miao, J. Liu, and L. Wang, “Using BODIPY Unit to Design Molecular Acceptors with Absorption Wavelength of >1000 nm for Organic Photodetectors,” CCS Chemistry 6 (2024): 2794–2803, <https://doi.org/10.31635/ccschem.024.202403865>.

[8]	Z. Lou, J. Tao, B. Wei, et. al., “Near-Infrared Organic Photodetectors toward Skin-Integrated Photoplethysmography-Electrocardiography Multimodal Sensing System,” *Advanced Science* 10 (2023): 2304174, <https://doi.org/10.1002/advs.202304174>.

[9]	J. W. Ha, A. Y. Lee, H. J. Eun, et. al., “High Detectivity Near Infrared Organic Photodetectors Using an Asymmetric Non-Fullerene Acceptor for Optimal Nanomorphology and Suppressed Dark Current,” *ACS Nano* 17 (2023): 18792–18804.

[10]	Y. Zhang, Y. Yu, X. Liu, et. al., “An n-Type All-Fused-Ring Molecule with Photoresponse to 1000 nm for Highly Sensitive Near-Infrared Photodetector,” *Advanced Materials* 35 (2023): 2211714, <https://doi.org/10.1002/adma.202211714>.

[11]	Z. Zhong, X. Liu, L. Li, et. al., “An Asymmetric A-D-π-A Type Non-Fullerene Acceptor Enables High-Detectivity Near-Infrared Organic Photodiodes,” *Science China Chemistry* 66 *(*2022): 242–250, <https://doi.org/10.1007/s11426-022-1385-8>.

[12]	Y. Zheng, Y. Chen, Y. Cao, F. Huang, Y. Guo, and X. Zhu, “Design of All-Fused-Ring Nonfullerene Acceptor for Highly Sensitive Self-Powered Near-Infrared Organic Photodetectors,” *ACS Materials Letters* 4 (2022): 882–890, <https://doi.org/10.1021/acsmaterialslett.2c00191>.

[13]	T. Liu, Z. Jia, Y. Song, et. al., “Near Infrared Self-Powered Organic Photodetectors with a Record Responsivity Enabled by Low Trap Density,” *Advanced Functional Materials* 33 (2023): 2301167, <https://doi.org/10.1002/adfm.202301167>.

[14] K. H. Huang, B. H. Jiang, H. C. Lu, et. al., “Electron-Rich Heptacyclic S,N Heteroacene Enabling C-Shaped A-D-A-type Electron Acceptors With Photoelectric Response beyond 1000 Nm for Highly Sensitive Near-Infrared Photodetectors,” *Advanced Science* 12 (2025): 2413045, <https://doi.org/10.1002/advs.202413045>.

[15]	Y. Xia, C. Geng, X. Bi, et. al., “Biomimetic Flexible High-Sensitivity Near-Infrared II Organic Photodetector for Photon Detection and Imaging,” *Advanced Optical Materials* 12 (2023): 2301518, <https://doi.org/10.1002/adom.202301518>.

[16]	L. Shao, L. Hong, Y. Cao, et. al., “Spiro-Conjugation in Narrow-Bandgap Nonfullerene Acceptors Enables Broader Spectral Response and Higher Detectivity for Near-Infrared Organic Photodetectors,” *Advanced Optical Materials* 11 (2023): 2202823, <https://doi.org/10.1002/adom.202202823>.

[17]	H. Zhang, R. Mao, L. Yuan, et. al., “Near-Infrared Organic Photodetectors with Spectral Response over 1200 nm Adopting a Thieno[3,4-c]thiadiazole-Based Acceptor,” *ACS Applied Materials & Interfaces* 16 (2024): 9088–9097, <https://doi.org/10.1021/acsami.3c15902>.

[18]	K. Cho, J. W. Ha, M. Nam, et. al., “Crystallization-Driven Optimization of Morphology and Performance in Near-Infrared Organic Photodetectors via Alkyl Side Chain Tuning of Narrow Bandgap Non-Fullerene Acceptors,” *Advanced Functional Materials* 34 (2024): 2400676, <https://doi.org/10.1002/adfm.202400676>.

[19] K. Valkeneers, J. Raymakers, Q. Liu, et. al., “A Tetrathienopyrrole-Based Ladder-Type Donor Polymer for High-Performance Organic Near-Infrared Cavity Detectors,” *Materials Horizons* 10 (2023): 5704–5711, <https://doi.org/10.1039/D3MH01010D>.

[20] J. Cong, Z. H. Huang, S. W. Liu, et. al., “Efficient SWIR Organic Photodetectors with Spectral Detection Extending to 1.4 µm Using a Benzobisthiadiazole-Based Acceptor,” *Small* 21 (2025): 2410418, <https://doi.org/10.1002/smll.202410418>.

[21] J. Zeng, Z. Ren, D. Lin, et. al., “Molecular-Device Co-Engineering of Ultra-Low Dark Current SWIR Organic Photodetectors for High-Quality Blood-Pressure Monitoring and Optical Communication,” *Advanced Materials* 37 (2025): 09923, <https://doi.org/10.1002/adma.202509923>.

[22] T. H. Kim, S. Y. Jeong, S. Oh, et. al., “Control of Chemical Doping-Mediated Space Charge for Energetic Band-Switching Modulation in Low-Noise Shortwave-Infrared Organic Photodetector,” *Advanced Materials* 37 (2025): 2500126, <https://doi.org/10.1002/adma.202500126>.

[23] X. Yu, H. Lin, Z. He, et. al., “Efficient Near-Infrared Organic Photodetectors with Spectral Response up to 1600 nm for Accurate Alcohol Concentration Detection,” *ACS Applied Materials & Interfaces* 15 (2023): 16918–16929, <https://doi.org/10.1021/acsami.2c22724>.

[24] S. Lee, J. Lee, H. R. Sim, C. So, and D. S. Chung, “Shortwave Infrared Organic Photodiodes Realized by Polaron Engineering,” *Advanced Materials* 36 (2024): 2310250, <https://doi.org/10.1002/adma.202310250>.

[25] N. Ghobadi, “ Band Gap Determination Using Absorption Spectrum Fitting Procedure,” *International Nano Letters* 3 (2013): 2, <https://doi.org/10.1186/2228-5326-3-2>.

[26] T. L. D. Tam, M. Lin, A. D. Handoko, and J. Xu, “Thermoelectric Performances of n-Doped Ladder-Type Conjugated Polymers Using Various Viologen Radical Cations,” *ACS Applied Polymer Materials* 3 (2021): 5596–5603, <https://doi.org/10.1021/acsapm.1c00920>.

[27] C.-H. Tsai, Y.-C. Lin, W.-N. Wu, S.-H. Tung, W.-C. Chen, and C.-L. Liu, “Optimizing the Doping Efficiency and Thermoelectric Properties of Isoindigo-Based Conjugated polymers using side chain engineering,” *Journal of Materials Chemistry C* 11 (2023): 6874–6883, <https://doi.org/10.1039/D3TC00883E>.

[28] W. Zhao, J. Ding, Y. Zou, C. A. Di, and D. Zhu, “Chemical Doping of Organic Semiconductors for Thermoelectric Applications,” *Chemical Society Reviews* 49 (2020): 7210–7228, <https://doi.org/10.1039/D0CS00204F>.

[29] J.-F. Ding, K. Yamanaka, S.-H. Hong, et. al., “Controlling the Thermoelectric Performance of Doped Naphthobisthiadiazole-Based Donor–Acceptor Conjugated Polymers through Backbone Engineering,” *Advanced Science* 11 (2024): 2410046, <https://doi.org/10.1002/advs.202410046>.

[30] Y.-H. Deng, D. Van, and T. Z. Hens, “Understanding and Equivalence of Response Time Measurements in Photodetectors,” *ACS Photonics* 13 (2026): 1752–1756, <https://doi.org/10.1021/acsphotonics.6c00438>.

[31] S. Wredh, M. Dai, K. Hamada, et. al., “Sb_2_Te_3_–Bi_2_Te_3_ Direct Photo–Thermoelectric Mid-Infrared Detection,” *Advanced Optical Materials* 12 (2024): 2401450, <https://doi.org/10.1002/adom.202401450>.

[32] J. Wang, Z. Xie, J. A. Liu, and J. T. Yeow, “Design of Room-Temperature Infrared Photothermoelectric Detectors Based on CNT/PEDOT:PSS Composites,” *Journal of Materials Chemistry C* 10 (2022): 15105–15113, <https://doi.org/10.1039/D2TC03159K>.

[33] E. Kaminski, N. Suleymanov, B. Minkovich, et. al., “Waveguide Integrated Self-Powered MoS_2_ Photodetectors in the Shortwave Infrared Wavelengths,” *ACS Photonics* 12 (2025): 6397–6405, <https://doi.org/10.1021/acsphotonics.5c01893>.

[34] X. He, F. Leonard, and J. Kono, “Uncooled Carbon Nanotube Photodetectors,” *Advanced Optical Materials* 3 (2015): 989–1011, <https://doi.org/10.1002/adom.201500237>.
